# Supplementary material for: Survival after Pure (Acute) Erythroid Leukemia in the United States: A SEER-Based Study
Source: Cancers (Basel). 2023 Aug 3;15(15):3941. doi: 10.3390/cancers15153941 (PMC10417752; doi:10.3390/cancers15153941)
Supplement: Supplementary file 1 [file cancers-15-03941-s001.zip › cancers-2450595-supplementary.pdf]

Supplementary Materials

Survival after Pure (Acute) Erythroid Leukemia in the United States: A SEER-Based study

Kriti Gera, Daniela Martir, Wei Xue and John R. Wingard

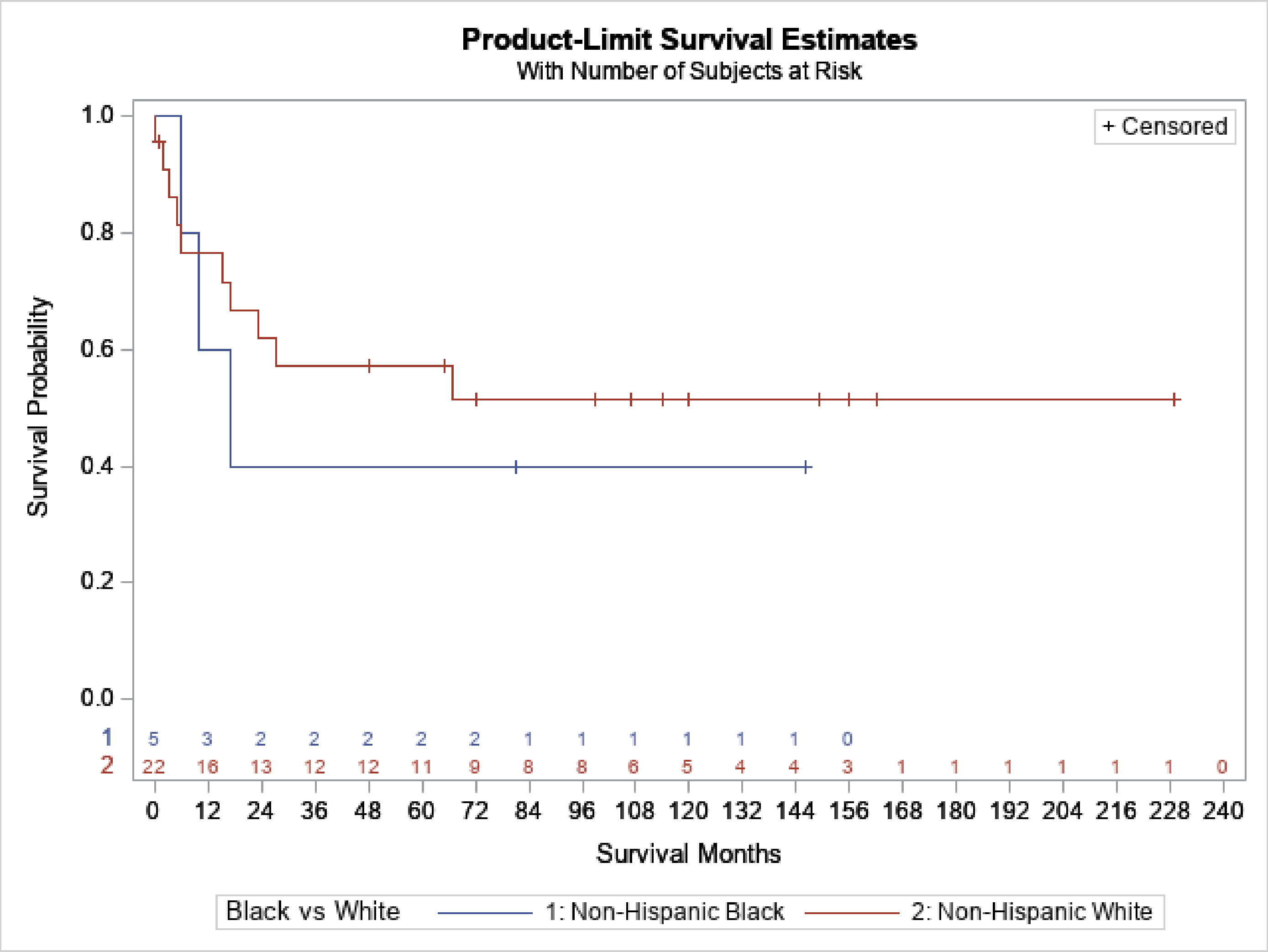

Figure S1. Overall Survival in children and adolescents based on race (p= 0.6536).

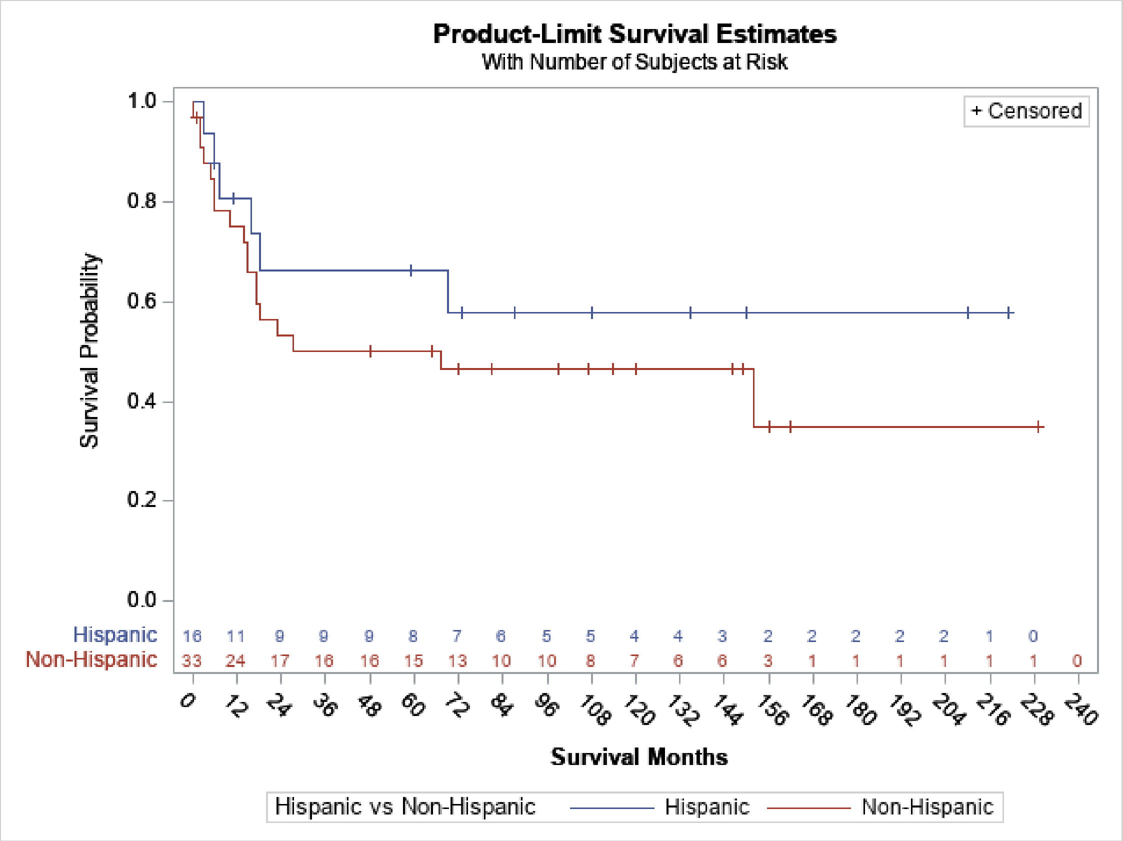

Figure S2. Overall Survival in children and adolescents based on ethnicity (p = 0.3385).

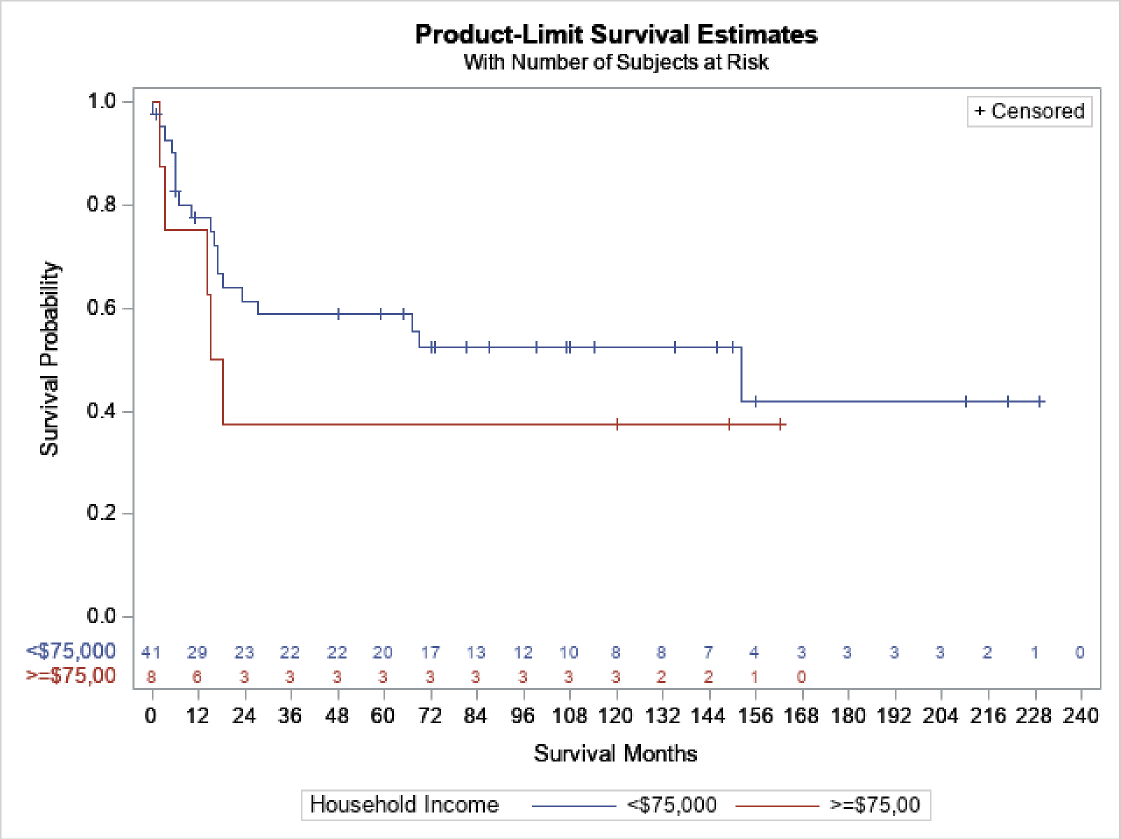

Figure S3. Overall Survival in children and adolescents based on median household income (p = 0.3807).

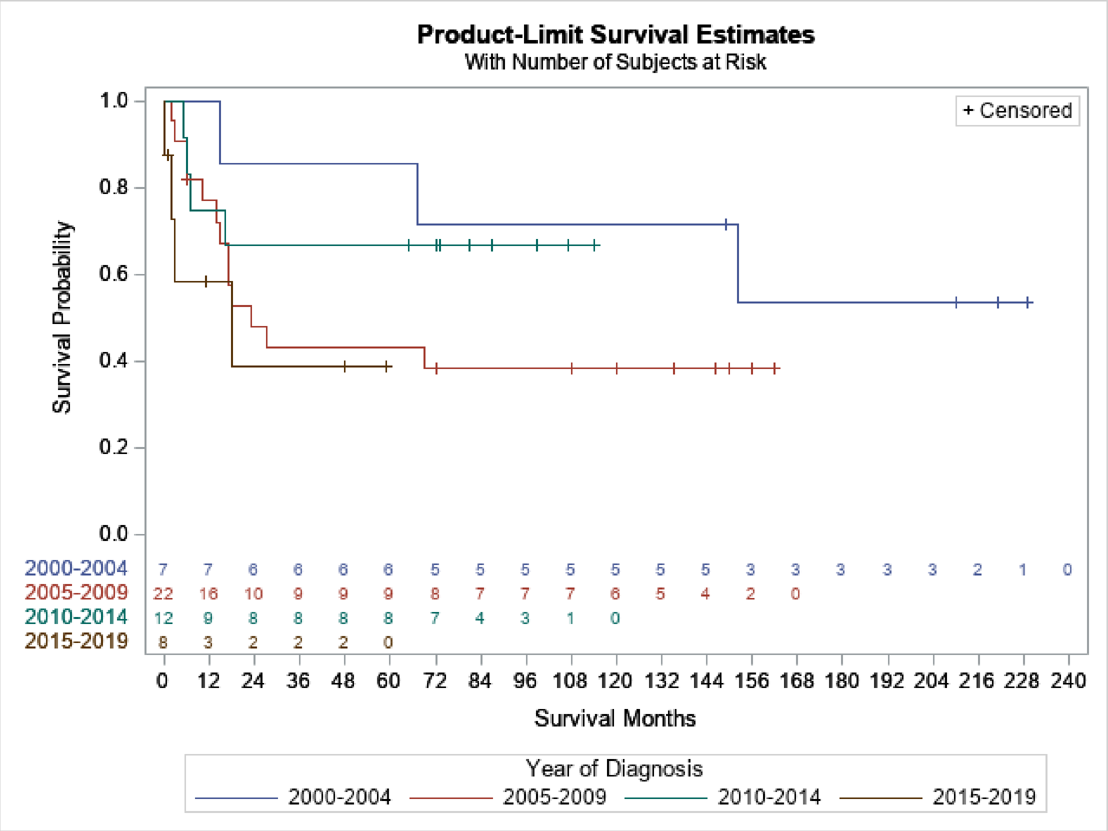

Figure S4. Overall survival in children and adolescents based on year of diagnosis ($p = 0.2650$).

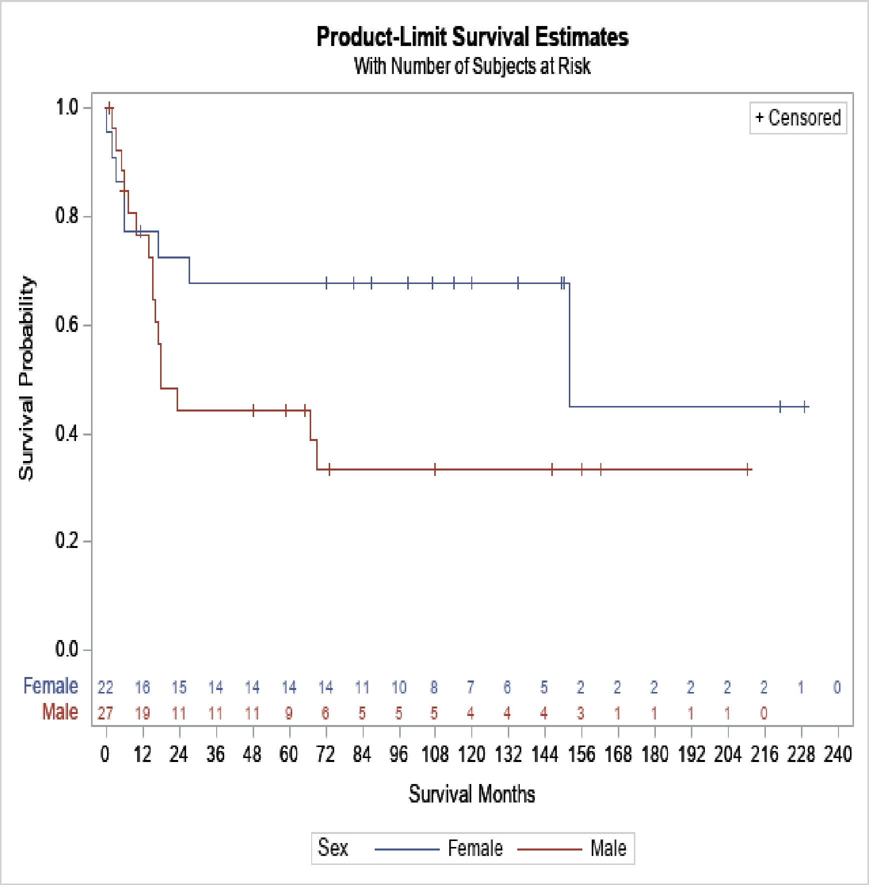

Figure S5. Overall Survival in children and adolescents based on gender ($p = 0.1111$).

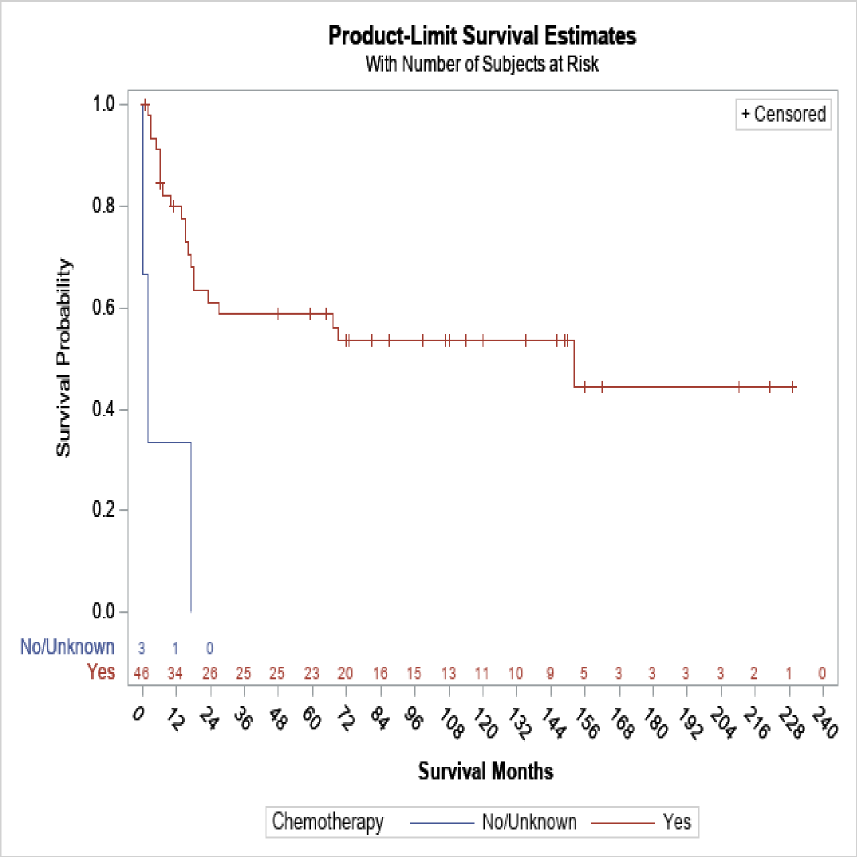

Figure S6. Overall Survival in children and adolescents based on treatment with chemotherapy (p = 0.0004).

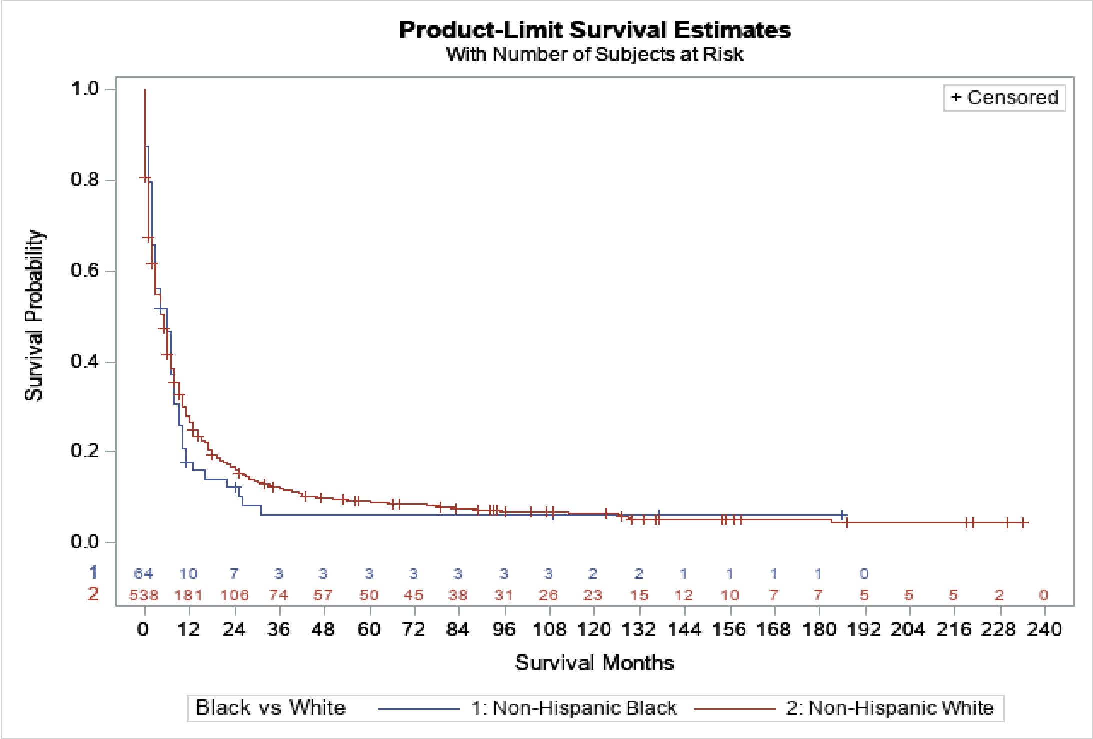

Figure S7. Overall Survival in adults based on race (p-value = 0.6970).

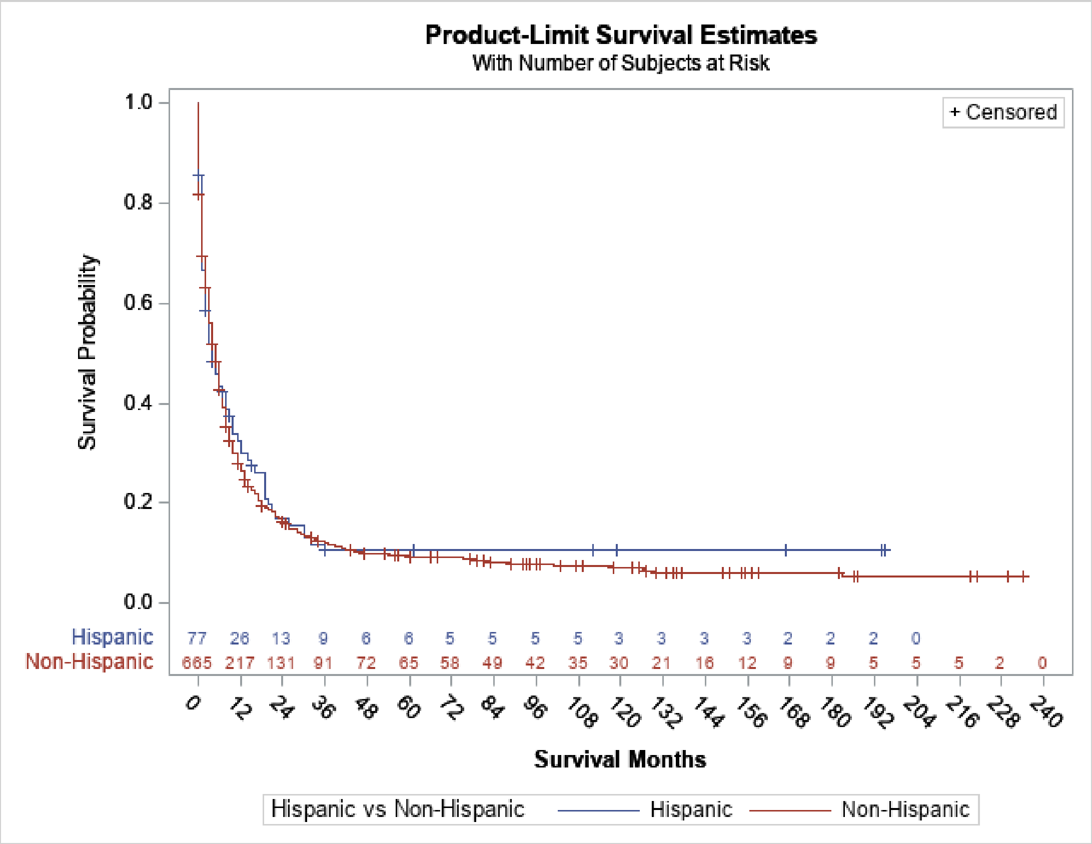

Figure S8. Overall Survival in adults based on ethnicity (p =0.6115).

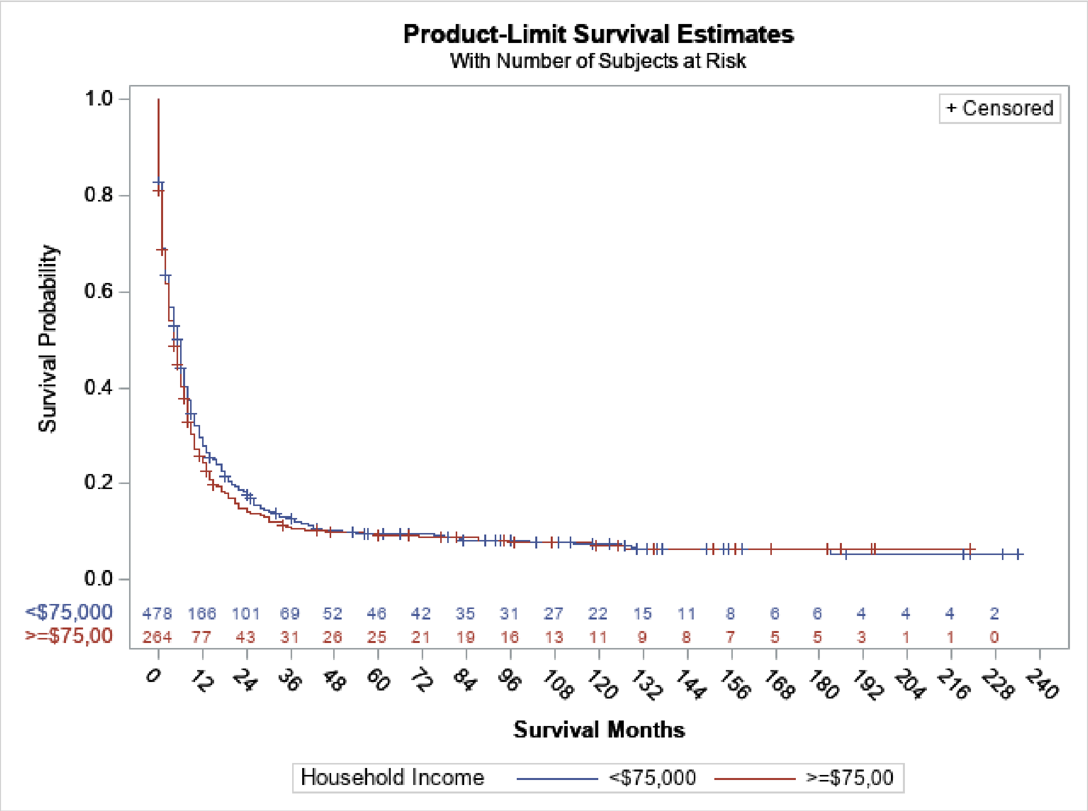

Figure S9. Overall Survival in adults based on median household income (p = 0.3320).

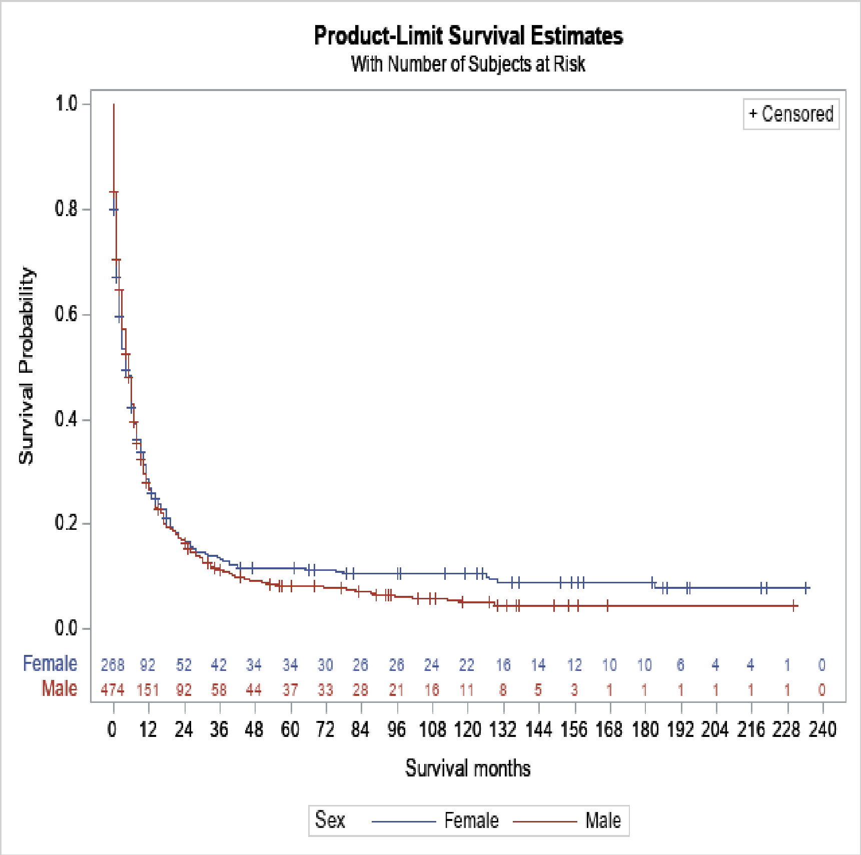

Figure S10. Overall Survival in adults based on gender (p = 0.5064).

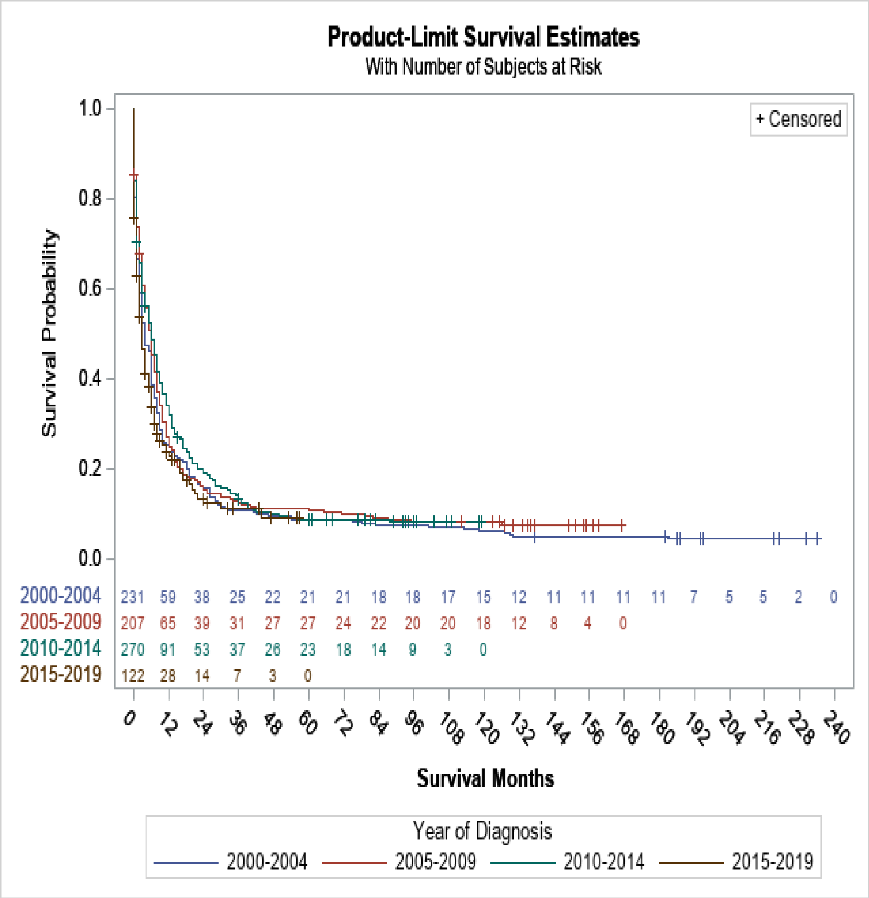

Figure S11. Overall Survival in adults based on year of diagnosis (p = 0.0824).
